# Development of a technique for psyllium husk mucilage purification with simultaneous microencapsulation of curcumin

**DOI:** 10.1371/journal.pone.0182948

**Published:** 2017-08-17

**Authors:** André Álvares Monge Neto, Rita de Cássia Bergamasco, Flávio Faria de Moraes, Antônio Medina Neto, Rosane Marina Peralta

**Affiliations:** 1 Postgraduate Program in Food Science, State University of Maringá, Maringá, Paraná, Brazil; 2 Department of Food Engineering, State University of Maringá, Maringá, Paraná, Brazil; 3 Department of Chemical Engineering, State University of Maringá, Maringá, Paraná, Brazil; 4 Department of Physics, State University of Maringá, Maringá, Paraná, Brazil; 5 Department of Biochemistry, State University of Maringá, Maringá, Paraná, Brazil; Kermanshah University of Medical Sciences, ISLAMIC REPUBLIC OF IRAN

## Abstract

This study focused on evaluating a technique for the psyllium husk mucilage (PHM) purification with simultaneous microencapsulation of curcumin. PHM was extracted with water and purified with ethanol. For the mucilage purification and simultaneous microencapsulation, an ethanolic solution of curcumin was used. After dehydration, the samples were analysed by instrumental techniques and evaluated for thermal stability. The presence of curcumin in the solution did not impair the yield of precipitated polysaccharide. Interactions of the dye and carbohydrates were confirmed by displacement of peaks in FT-IR and FT-Raman spectroscopy. The onset temperature of degradation of microcapsules was superior to that of curcumin. Thermal stability in solution at 90°C also improved. After 300 minutes of heating, the microcapsules had a remnant curcumin content exceeding 70%, while, in standard sample, the remaining curcumin content was 4.46%. Thus, the developed technique was successful on purification of PHM and microencapsulation of curcumin.

## Introduction

Curcumin is the main phenolic dye extracted from turmeric. This dye has low water solubility and low stability characteristics that make its use in food products unfeasible [1]. Microencapsulation of curcumin solves these problems while retaining its antioxidant, antibiotic and antifungal properties [2] [3] and its ability to hamper the proliferation of cancer cells [4] as well as improving its bioavailability and ability to aid healing [3].

Microencapsulation technique protects solids, liquids or gases from environmental factors such as oxygen, light, and moisture and releases them in specific conditions [5] [6]. This technology may lead to benefits such as increased stability during storage and improve the water solubility of colorant compounds [6].

Microcapsules may have different dimensions and shapes. This variation is due the encapsulating agent used and the encapsulation process conditions [7]. In order to be successfully applied as an encapsulating agent, a substance must have characteristics such as low cost, easy handling and good rheological properties at high concentrations. The substance must protect the core material during production and throughout the storage period without reacting with it. Furthermore, it must disperse or emulsify the guest molecule and stabilise the emulsion. Thus, few materials have characteristics that justify their application in industrially compatible technologies for microencapsulation [5] [6] [8]. Freeze drying and spray drying are very common processes used to produce microcapsules and, because of their differences on operation, final products may have different characteristics such yield of microencapsulation and degradation of internal molecule [9] [10].

Studies with natural mucilage have shown interesting properties that justify their potential use as encapsulating agents: nopal mucilage was successfully used to produce microcapsules of gallic acid [11] and betacyanins [12] and *Aloe vera* gel has been used as an encapsulant of the probiotic bacteria *Weissella confusa* maintaining 80% cell viability, much higher than a control treatment with sodium caseinate (21.666%) [13].

Hydrocolloid extracted from psyllium husk is compound by a complex heteropolysaccharide, arabinoxylan, and it has characteristics which justify its use in many areas [14]. Psyllium husk mucilage, with added glycerol, may be used in the production of films [15]. Psyllium hydrocolloid is considered a good flocculant agent in wastewater treatment because it is inexpensive, readily available and biodegradable [16]. It also showed good performance as a suspending agent for paracetamol and in tableting, with good bonding properties [17]. The use of psyllium mucilage to stabilise emulsions was succeed: low concentrations of this polysaccharide on yoghurt formulations (0.63%) induced the lowest syneresis [18] and psyllium mucilage was used to stabilise a microbial canthaxanthin emulsion, achieving 15 days of dye stability [19]. Also, psyllium husk mucilage has activities in intestinal regulation, preventing constipation, diarrhea, irritable bowel syndrome, reducing glucose in the post-prandial period and lowering cholesterol [20]. In addition, there was not found papers using psyllium husk mucilage as encapsulating agent of colorants. Therefore, this study aims to evaluate the use of psyllium husk mucilage as encapsulating agent of curcumin by developing a process of psyllium mucilage purification with simultaneous encapsulation of the coulorant. Additionally, the microcapsules were analysed by instrumental techniques and evaluated for thermal stability.

## Materials and methods

### Materials

Psyllium husk was acquired in local markets of Maringa, Brazil. Curcumin was purchased from Sigma Aldrich® (purity above 65% as reported by the manufacturer). Ethanol and other reagents used were of analytical grade.

### Methods

#### Mucilage extraction

Psyllium husk mucilage (PHM) was extracted by mixing with water in a 1:100 (w/v) ratio, for 1.2 h, under constant agitation at 80°C. The sample was filtered through cloth for mucilage separation and stored at 4°C for later use [18].

#### Mucilage purification and microcapsule production

PHM was stirred for 10 minutes with ethanol at a ratio of 100:75 (mucilage volume:ethanol volume). After stirring, the mixture was centrifuged at 2000 rpm for 3 minutes at room temperature. The supernatant was used to quantify the non-precipitated carbohydrates. Ethanol was removed from the precipitate by using a rotary evaporator. Spray drying and freeze drying techniques were used to dehydrate the carbohydrates.

In order to produce the microcapsules, ethanol was replaced by an ethanolic solution of curcumin at the same ratio as above (100:75 –mucilage volume:solution volume). A curcumin solution was prepared in order to maintain a 1:20 ratio of dye to polysaccharide, as determined by preliminary tests. The mixture was centrifuged at 2000 rpm for 3 minutes at room temperature and the supernatant was used for analysis of the efficiency of the purification process and quantification of the curcumin not loaded into the mucilage. Ethanol was removed from the microcapsules using a rotary evaporator. Subsequently the microcapsules were spray dried or freeze dried.

A B-191 mini spray dryer (Büchi–Switzerland) was used, with an inlet temperature of 175°C, an outlet temperature between 102 and 105°C, and a sample injection flow rate of 10 mL.min^-1^. Microcapsule recovery after spray drying processes was determined by Eq 1 [21].

$$Microcapsule\;recovery=\frac{mass\;of\;microcapsule}{mass\;of\;raw\;material}$$ (1)

The freeze drying process was carried out in an Alpha 1–4 LD apparatus, for 48 hours at a temperature of -45°C. The samples were frozen in liquid nitrogen.

Moisture [22] and carbohydrate [23] analyses were performed on the raw psyllium husk gum and on carbohydrates precipitated with ethanol and with curcumin solution. The remaining solids on supernatant after precipitation were quantified dehydrating 20 mL of supernatants at 105°C and subsequently weighing the residue [16].

The curcumin contents of the supernatants were quantified by measuring the absorbance at 425 nm, in a spectrophotometer compared to a curcumin standard curve. The content of curcumin loaded into PHM was calculated by Eq 2.

$$Curcumin\;loaded=\frac{Curcumin\;concentration\;in\;supernatant}{Original\;curcumin\;concentration}$$ (2)

#### Quantification of microencapsulated curcumin

To accomplish the quantification of curcumin, the methodology described by Mangolim et al. [24] was used, with some modifications. Initially, 10 mg of each microcapsule preparation was diluted with 3 mL of distilled water. The mucilage was precipitated by the addition of 5 mL ethanol and subsequent centrifugation at room temperature for 3 minutes at 2000 rpm. The supernatant was reserved and this procedure was repeated four times, with a total of 20 mL of ethanol added. The supernatant was homogenised and its absorbance was measured in a spectrophotometer at 425 nm. Loading ability of PHM was calculated using the Eq 3 [24].

$$Loading\;ability=\frac{mass\;of\;curcumin}{mass\;of\;encapsulating\;agent}.100$$ (3)

Microcapsules and control samples were stored at -18°C until characterisation by instrumental techniques and thermal stability.

#### Microcapsule morphology–scanning electron microscopy (SEM)

All samples were metallised in a model SCD 050 –BOL-TEC Sputtering instrument and the images were taken in a Shimadzu SS-550 scanning electron microscope.

#### Thermal analysis—DSC and TGA

Thermogravimetric analysis (TGA) and differential scanning calorimetry (DSC) were performed using a NETZSCH STA 409 PG instrument linked to a NETZSCH TASC 424/4 controller (Germany). The nitrogen flow rate was 30 cm³ min^-1^ and the heating rate was 10°C min^-1^ starting at 20°C until 600°C, with a platinum capsule [25].

#### X-Ray Diffraction (XRD)

X-ray diffractograms of all samples were obtained using a Shimadzu model LabX XRD-6000 diffractometer under environmental conditions in the range of 10–80° (2θ) [16].

#### FT-IR and FT-Raman spectroscopy

FT-IR analysis was performed using a Model Vertex 70v infrared Fourier transform spectrometer (Bruker, Germany) using a resolution of 4 cm^-1^ in the range 400 to 4000 cm^-1^. All presented values are an average of 120 scans. The FT-Raman spectra were obtained using the same spectrometer with a Ram II accessory and the presented values are an average of 500 scans. Standards of curcumin, PHM, microcapsules, and simple mixture samples were analysed using these techniques. Simple mixture samples were prepared by adding curcumin to freeze dried or spray dried PHM aiming to maintain the proportions obtained in section “Quantification of microencapsulated curcumin”.

#### Thermal stability

Samples of 10 mg of the microcapsules and standard curcumin were maintained at temperatures of 100, 150 and 200°C. Samples were collected after 0, 30, 60 and 120 minutes in order to quantify the remaining curcumin content according to “Quantification of microencapsulated curcumin” [26].

In order to evaluate the stability of the curcumin solution, microcapsules (0.1%) were solubilised in 100 ml of water. The temperature was maintained at 90°C for 300 minutes. Aliquots were taken at 0, 15, 30, 45, 60, 90, 120, 150, 180, 240 and 300 minutes and cooled in an ice bath to 25°C [27]. In order to quantify the remaining dye, 1 mL of the cooled sample was washed three times with ethanol and centrifuged, with a total of 10 mL of solvent. The supernatant was homogenised and its absorbance was measured at 425 nm in a spectrophotometer. A control sample was prepared by solubilising the equivalent amount of microencapsulated curcumin in an aqueous solution of Tween 80 (0.2%) [28].

#### Statistical analysis

The assays were performed in triplicate. Analysis of variance (ANOVA) and all figures were constructed using Origin 6.0® software.

## Results and discussion

### Mucilage purification and microcapsule production

Results show that curcumin solution was as effective as pure ethanol for purifying PHM (Table 1). The moisture and carbohydrate contents differed significantly before and after the purification process, and the presence of curcumin did not affect the process, resulting in mucilages with similar characteristics and yields.

**Table 1:** Characterisation of the samples.

	*Crude mucilage*	*Ethanol purified mucilage*	*Curcumin+ethanol purified mucilage*
Moisture (%)	99.24^a^ ± 0.02	98.41^b^ ± 0.05	98.31^b^ ± 0.10
Total carbohydrates (%)	0.89^b^ ± 0.01	2.27^a^ ± 0.09	2.19^a^ ± 0.12
Precipitated solids (%)	-	74.68^a^ ± 1.25	73.91^a^ ± 3.95

^a,b^ Numbers with the same superscript letters in the same line are not different at 5% significance (p<0.05).

By quantification of the curcumin remaining in the supernatant it was determined that 56.02 ± 1.6% of the dye present in the initial solution was precipitated with carbohydrates present in PHM. This result is in agreement with that obtained by Mishra et al. (2002), which used psyllium mucilage for the removal of suspended solids in tannery effluents, without pH modification, with a maximum removal of suspended solids of 57.89% [16].

Using Eq (3) to process the experimental data, the loading ability of PHM was 20.72 mg.g^-1^ and 22.91 mg.g^-1^ for freeze dried microcapsules and spray dried microcapsules, respectively. Curcumin microencapsulation with soluble starch presents very similar values of loading ability (6.32 to 27.86 mg_Curcumin_/g_starch_) to ones obtained experimentally [24]. Both techniques, spray drying and freeze drying, provided microcapsules with similar contents of curcumin. Nevertheless, spray drying process had microcapsule recovery yield of 10.21%, a very low value comparing with other works which used this technique to encapsulating natural compounds [29] [21].

### Microcapsule morphology–scanning electron microscopy (SEM)

**Fig 1:**
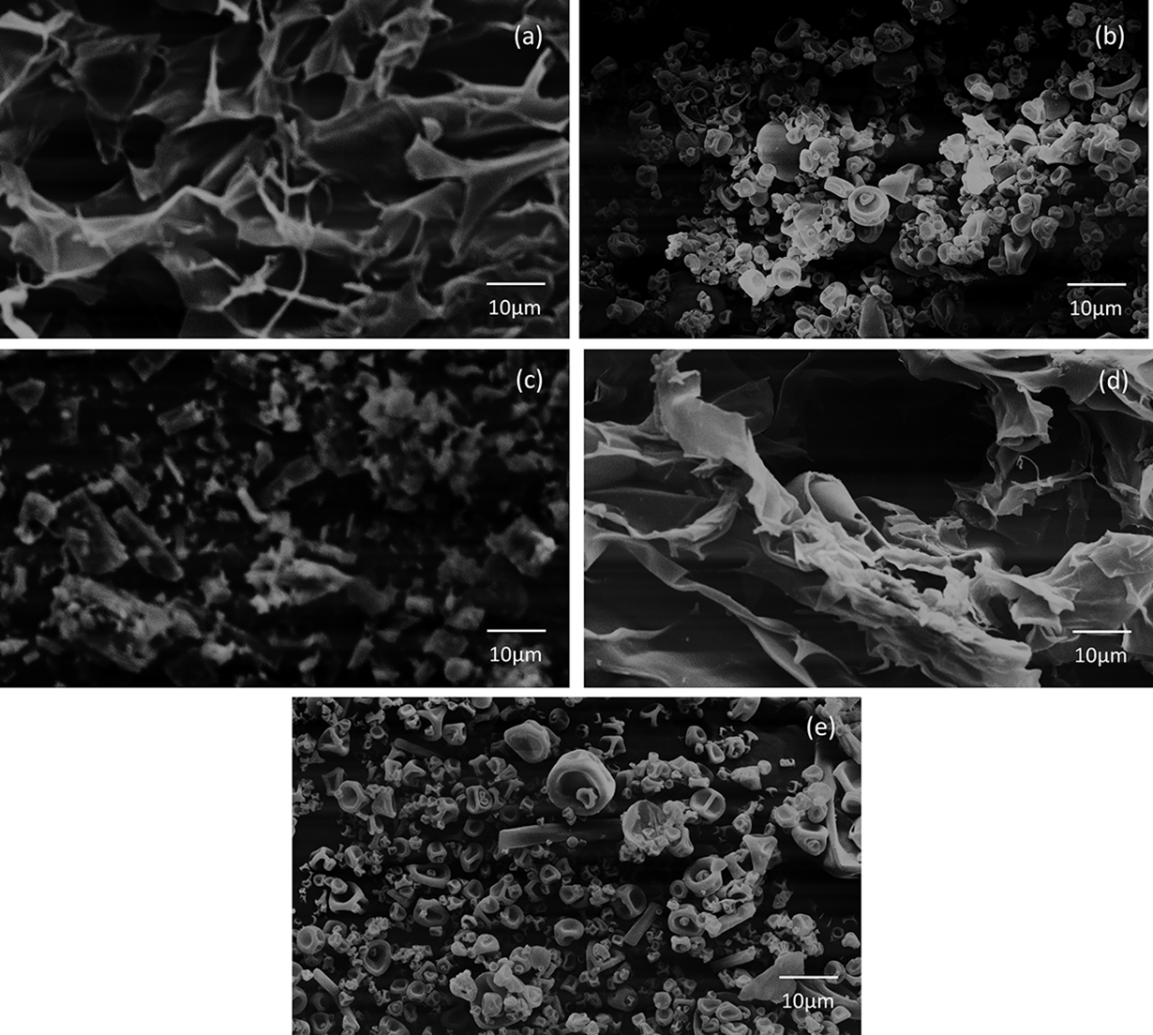
SEM images: a) Freeze-dried mucilage; b) Spray-dried mucilage; c) Curcumin; d) Freeze-dried microcapsule and e) Spray-dried microcapsule.

The differences in morphology observed in Fig 1 are due to the different drying processes [30]. The porous appearance presented by freeze-dried samples is due to ice crystal sublimation [31]. Spray-dried samples have rounded structures with or without concavities on the surface. These structures are common in spray-dried samples, and they occur due to the rapid evaporation of water droplets [32].

### Thermal analysis—DSC and TGA

**Fig 2:**
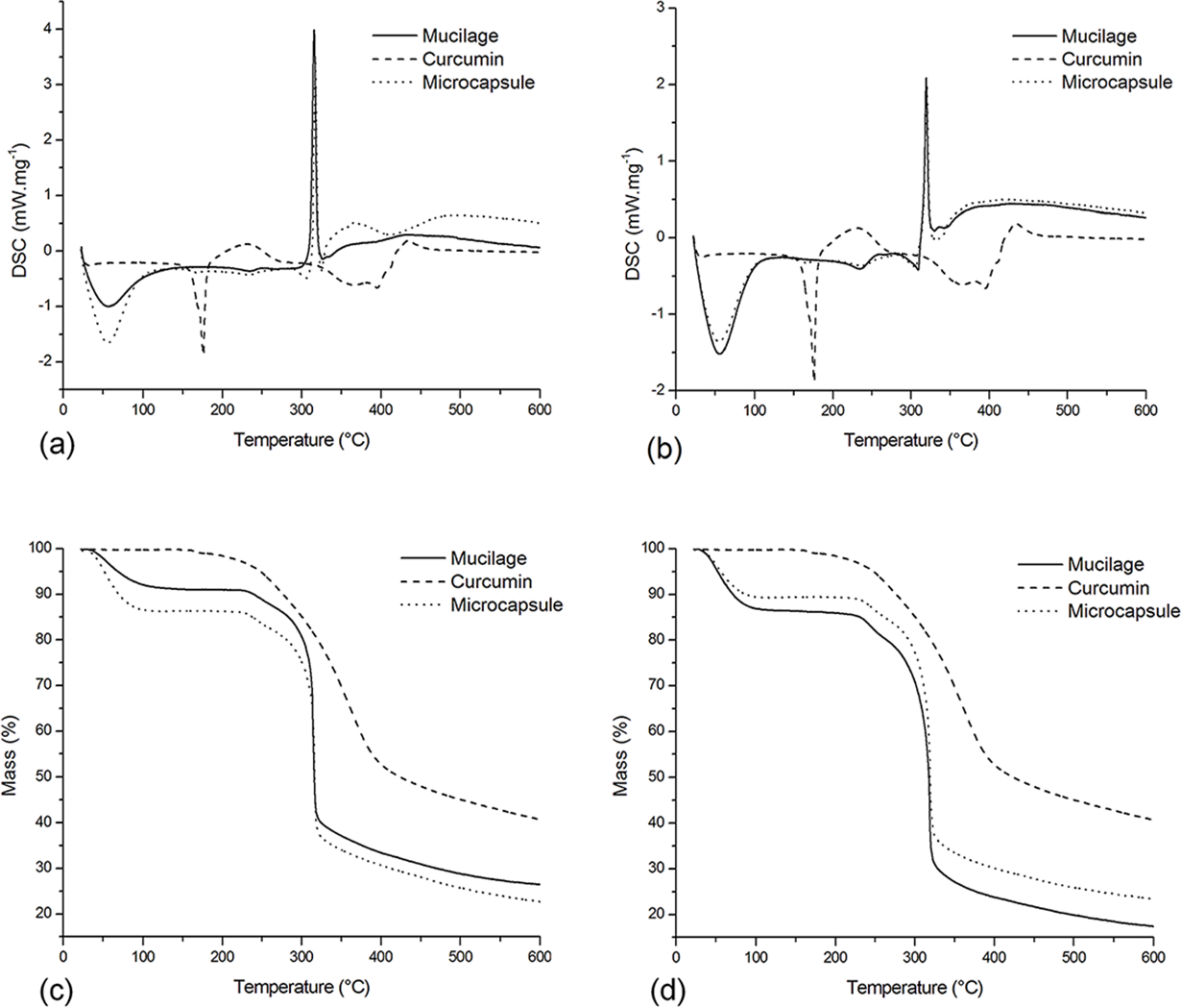
Thermal analysis of PHM, curcumin and microcapsules. (a) DSC results for freeze dried samples; (b) DSC results for spray dried samples; (c) TGA results for freeze dried samples and (d) TGA results for spray dried samples.

Thermal analysis presented differences in release energy patterns and thermal degradation common to microencapsulated materials (Fig 2).

As a hydrophobic molecule, the curcumin curve presented no loss of mass until 104°C (TGA) and no endothermic peak in this region (DSC), which is characteristic of water loss. Mucilages and microcapsules, which are composed largely of arabinoxylans, hydrophilic polysaccharides, exhibit weight loss and endothermic peaks that characterise the evaporation of water during the analysis. The thermograms obtained in present work are in accordance to those described by Marcolino et al. [33] and Iqbal [25], authors who studied curcumin and arabinoxylans, respectively. Therefore, the mass losses obtained by these authors are very similar to those described in this work.

Endothermic peaks near 176°C can be observed in the curcumin DSC pattern and less intensely in the microcapsule DSC patterns. These peaks indicate the melting point of free curcumin and/or curcumin at the surface of microcapsules. Exothermic peaks around 230°C in the curcumin pattern are probably due to decomposition of the dye [34]. In the thermogram, curcumin mass loss starts at around 180°C. For microcapsules the mass loss started at 223°C and exothermic peaks were observed only near to the degradation temperature of PHM. These patterns indicate that PHM improved the thermal stability of the dye.

### XRD

**Fig 3:**
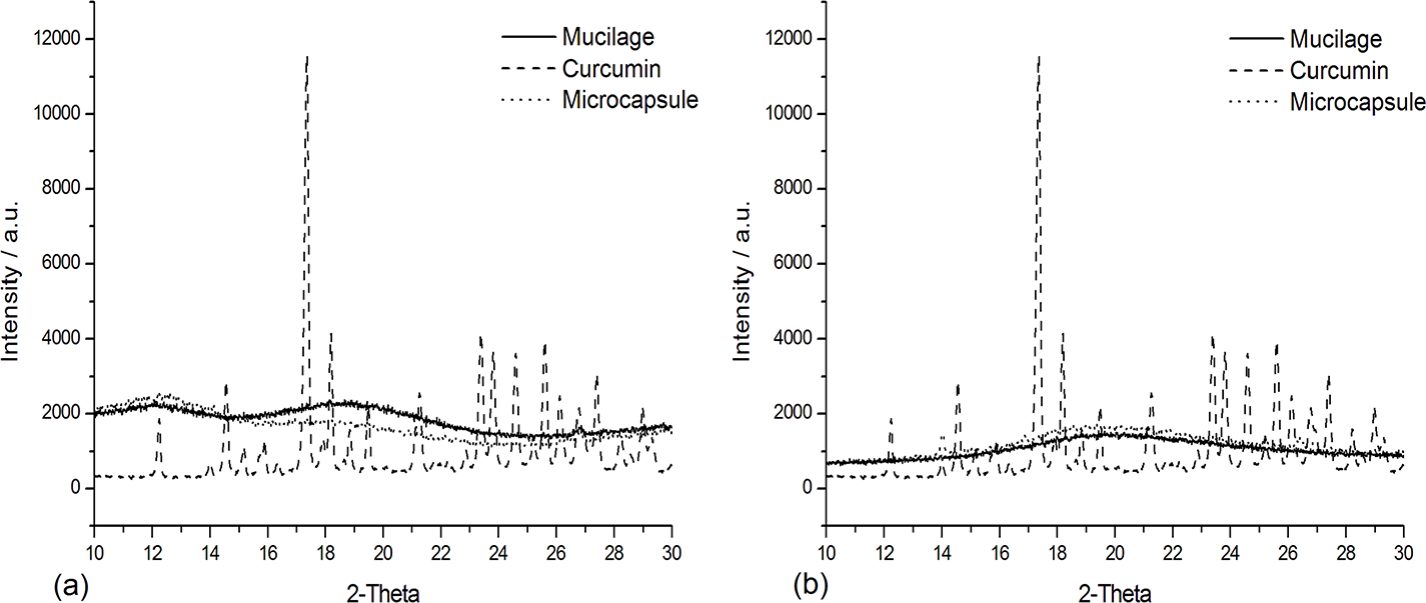
XRD patterns of (a) freeze dried samples and (b) spray dried samples.

The X-ray diffractograms are shown in Fig 3. PHM samples presented amorphous characteristics. Curcumin, a crystalline molecule with well-defined peaks, presented a modified diffractogram pattern, showing amorphous behaviour, after the PHM microencapsulation process. Mishra et al (2002) observed similar behaviour in diffraction pattern of PHM before and after its use in the treatment of domestic and tannery effluents [16]. This change in diffraction patterns indicates the involvement of curcumin molecules with PHM [24]. The microcapsules obtained by both techniques, spray-drying and freeze-drying, presented amorphous patterns. The absence of characteristic crystalline peaks of curcumin in the microcapsules obtained by both techniques reinforces their efficiency in protecting this natural dye.

### FT-IR spectroscopy

**Fig 4:**
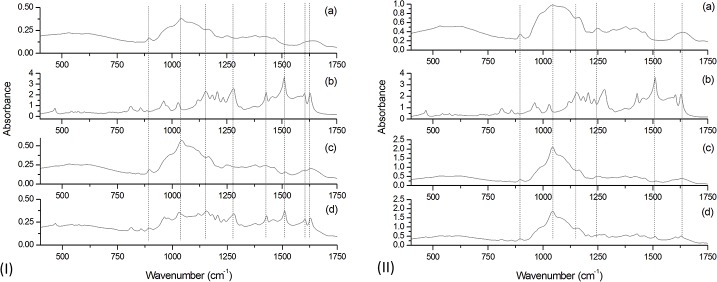
FT-IR spectra of (I) freeze dried samples and (II) spray dried samples: (a) PHM; (b) Curcumin; (c) Microcapsule; (d) Simple mixture.

FT-IR absorbance spectra are shown in Fig 4 and Table 2 lists the characteristic peaks of arabinoxylans and curcuminon FT-IR and FT-Raman spectra.

**Table 2:** FT-IR Peak assignment of arabinoxylans [35] and curcumin [36] and FT-Raman assignment peaks of curcumin [37].

FT-IR assignment peaks	PHM Arabinoxylans	1000–1200	Characteristic band of arabinoxylans
		~1044	(C–OH) bending
		1500–1700	Proteins and aromatic rings
		1539	Amide II
		1654	Amide I
	Curcumin	856	γ(CH) of aromatic rings and skeletal CCH
		1510	ν(C = O), δ(CCC) and δ(CC = O)
		1602	ν(C = C) of aromatic rings
		1627	ν(C = C) and ν(C = O) of inter-ring chain
FT-Raman assignment peaks	Curcumin	1151	δ(CCH) of aromatic rings and δ(C–OH) and δ(C–OH) of the enolic group coupled to δ(C = CH) in the inter-ring chain
		1250	δ(CH) of aromatic rings combined to ν(C–O) of the ether groups
		1317	δ(CCH) of inter-ring chain C10 and C11
		1430	δ(CCC), δ(CCH) and δ(C–OH) of aromatic rings
		1600	ν(C = C) of aromatic rings
		1627	ν(C = C) and ν(C = O) of inter-ring chain

The curcumin spectrum presents peaks that confirm its characteristic structure (Fig 4I and 4II–b), at 856 cm^-1^, 1510 cm^-1^, 1602 cm^-1^ and 1627 cm^-1^. The PHM spectrum includes peaks that indicate that the extracted mucilage is composed by arabinoxylans, such as the large ring between 1000 and 1200 cm^-1^ and the peak at 1045 cm^-1^ [35] [14].

Comparing the spectra of curcumin and freeze dried microcapsules (Fig 4–I.b-c, respectively) it can be noted that peaks assigned to the coulorant had lower intensity (1510 cm^-1^ and 1627 cm^-1^) or were not observed (856 cm^-1^ and 1602 cm^-1^) when microencapsulated. In the microcapsule spectrum, the large ring characteristic of arabinoxylans (1000–1200 cm^-1^) can be seen with its peak at 1045 cm^-1^. The simple mixture pattern (Fig 4 –I.d) exhibits characteristics of both PHM and curcumin. The difference in patterns between microcapsules and simple mixtures indicate the formation of hydrogen bonds between phenolic compounds and wall material polymers [24].

The spray dried microcapsule pattern (Fig 4 –II.c) exhibits changes compared with the coulorant peaks, including lower intensity and extinction of characteristic peaks. However, the simple mixture pattern was not the sum of patterns as expected (Fig 4 –II.d) making it difficult to prove the interaction between the dye and PHM. The decrease in the intensity of the FT-IR peaks alone does not provide conclusive information regarding the production of the microcapsules [26]. Combined FT-IR and FT-Raman data may help to elucidate the formation of the microcapsules [38].

### FT-Raman spectroscopy

**Fig 5:**
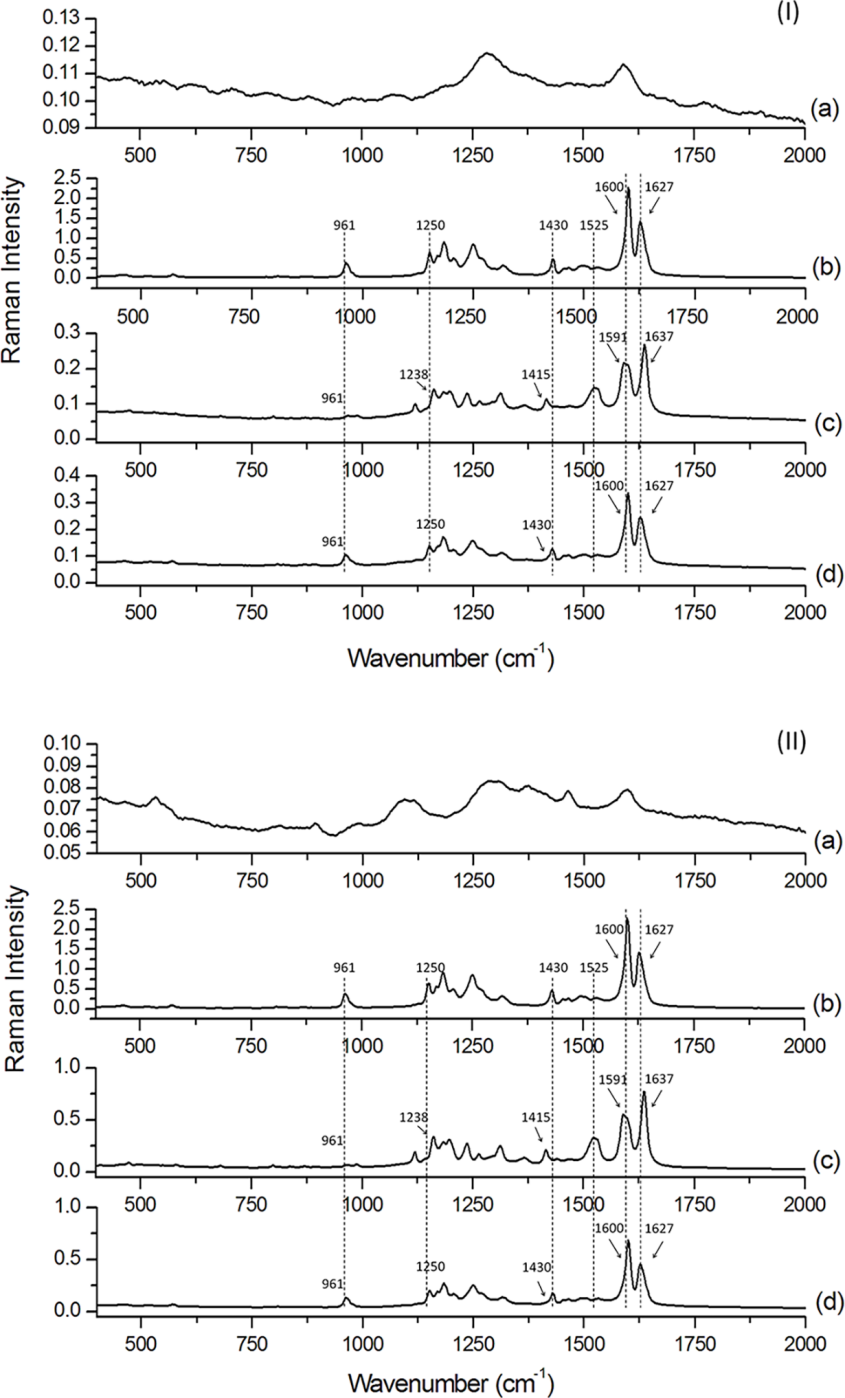
FT-Raman spectra of (I) freeze dried samples and (II) spray dried samples: (a) PHM; (b) Curcumin; (c) Microcapsule; (d) Simple mixture.

Analysis of the FT-Raman spectra shown in Fig 5 may help in elucidating the regions of the curcumin molecules protected within the microcapsule. As the peak intensity of freeze dried and spray dried PHM (Fig 5I and 5II.a) was lower than in other samples, they were not used in order to attest microcapsule formation. Curcumin assigned FT-Raman peaks are displayed in Table 2.

Curcumin presented its characteristic peaks (Table 2 and Fig 5I and 5II–b) and, after the process of microencapsulation in PHM, these characteristic peaks shifted to different values. These displacements indicate that every region of the dye is interacting with PHM in the wall of the microcapsules by the formation of hydrogen bonds. In addition, the peak arisen in 1525 cm^-1^ (Fig 5I and 5II–c) indicate an interaction not common to non-encapsulated curcumin. Mangolim et al. [26] analysed the inclusion complex curcumin-β-cyclodextrin formation by FT-Raman spectroscopy. The spectrum obtained by these authors presented a similar peak in curcumin complexed to cyclodextrin at 1523 cm^-1^ which was not found with free curcumin. Simple mixture samples exhibited spectra very similar to curcumin, without significant changes and, as the intensity of PHM peaks was much smaller than intensity of the curcumin peaks, they were not visible in the spectrum of the simple mixture. These results indicate that purification process of psyllium mucilage with simultaneous microencapsulation of curcumin is able to promote interaction of the coulorant molecule with the polysaccharide of the mucilage. In addition, the combined analysis of the spectra obtained by FT-IR and FT-Raman confirm the interaction of PHM with most or almost most parts of the molecule of curcumin in both microencapsulation techniques.

### Thermal stability

**Fig 6:**
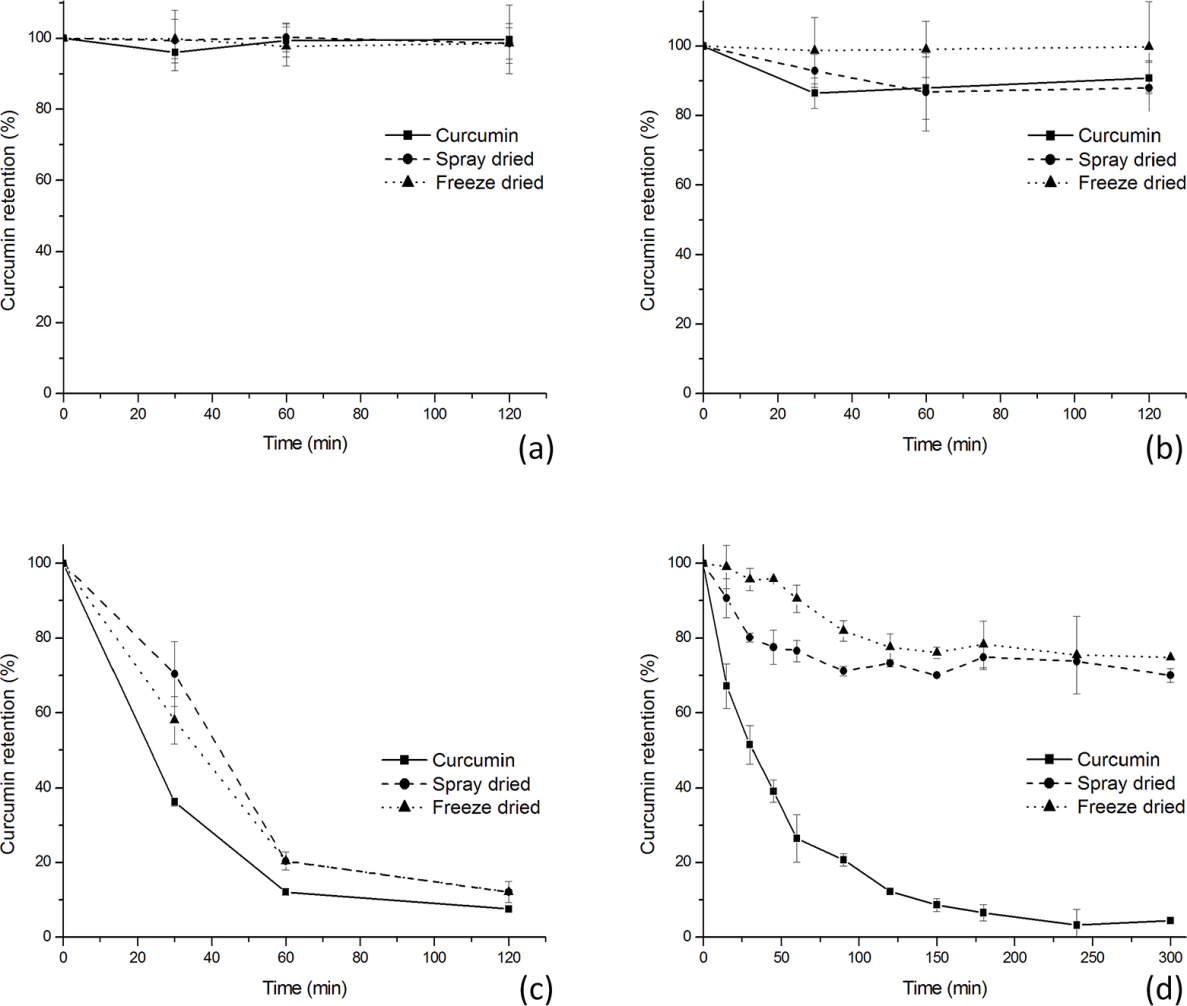
Curcumin and microcapsule preparations stability. (a) powder stability at 100°C; (b) powder stability at 150°C; (c) powder stability at 200°C; (d) solution stability at 90°C.

The percentage of curcumin remaining after 120 minutes of exposure to temperatures of 100, 150 and 200°C is shown in (Fig 6A–6C). The microcapsules obtained from spray drying and freeze drying techniques presented the same behaviour in these assays, maintaining similar contents of curcumin after the thermal exposure. Microcapsules were more stable than curcumin in the first 30 minutes at 150 and 200°C. Comparing these data to the TGA results (Fig 2(C) and 2(D)), it is notable that curcumin begins to degrade quite substantially at 100°C, this explains the greater decay of unencapsulated dye at the beginning of the experiment at higher temperatures. Prolonged exposure of microcapsules to these temperatures causes degradation of the dye, resulting in the remaining curcumin content being similar to that of pure dye. Mangolim et al. (2014), in a similar test, observed the same behaviour comparing curcumin to its complex with β-cyclodextrin [26].

The use of PHM as a gelling agent generally requires the use of elevated temperatures around 85–90° C [18] [39]. Thus, to evaluate dye stability under gelation conditions it is necessary to predict the feasibility of the use of PHM as a wall material for encapsulating curcumin.

The decay curves measuring curcumin content in solution at 90°C (Fig 6D) indicate that microcapsules make the dye more stable to exposure to elevated temperature. After 300 minutes, the control sample showed remaining dye content of less than 5% of the initial value. PHM encapsulated curcumin on the other hand, maintained a dye content of more than 70% of the initial value. In the first fifteen minutes, the freeze dried sample showed a minimal loss of curcumin, indicating that is possible to expose the microcapsules to high temperatures to allow the complete dissolution of PHM without substantial dye losses. The process used to produce microcapsules influenced curcumin stability in this assay; samples obtained from spray drying technique were less stable than samples obtained from freeze drying. Cano-Higuita, Malacrida and Telis [30] observed the same behaviour when they studied curcumin microcapsules obtained with different wall materials by freeze-drying and spray-drying. The authors observed fast degradation of the spray-dried microcapsules when under light storage. These authors attributed this behaviour to the higher rates of degradation of the curcumin that remained on the spray-dried microcapsule surface.

Liu et al. [40]produced curcumin microcapsules with whey protein isolated by spray-drying at 110 and 150°C. These authors quantified curcumin by its absorbance at 425 nm, similarly to what was done in this study, and evaluated the antioxidant activity of the microcapsules. In their conclusions, they stated that the curcumin located inside the microcapsules was effective in donating hydrogen atoms to the DPPH (2,2-diphenyl-1-picrylhydrazyl) radicals. Thereby, the microcapsules produced in the present work have potential antioxidant activity under food processing conditions. However, this hypothesis needs to be confirmed in future work.

## Conclusion

PHM arabinoxylan precipitation promotes incorporation of 56% of the curcumin in solution. It interacts with the dye and consequently improves the coulorant thermal stability. The presence of the dye did not affect the yield of precipitated arabinoxylan. Structural changes resulting from polysaccharide interactions with curcumin were confirmed by alterations in FT-Raman spectra when comparing the pure substances and the microcapsules. PHM microencapsulated curcumin had improved thermal stability as shown by thermal analysis (DSC and TGA) and by exposure to high temperatures. Freeze dried microcapsule had higher thermal stability than the spray dried one and, allied to low microcapsule recovery of spray drying technique, freeze drying process are more advantageous. Thus, PHM are efficient in protecting curcumin to thermal degradation and, as it is a non-toxic and biodegradable mucilage, also has potential to be used in food processing as a gelling and couloring agent.
